# Diagonal Earlobe Crease, a Marker of Coronary Artery Disease: A Case Report on Frank's Sign

**DOI:** 10.7759/cureus.4219

**Published:** 2019-03-11

**Authors:** Araz Baboujian, Prema Bezwada, Cesar Ayala-Rodriguez

**Affiliations:** 1 Cardiology, The Brooklyn Hospital Center - Mount Sinai Heart, Brooklyn, USA

**Keywords:** case report, diagonal earlobe crease, frank sign, physical examination, coronary atery disease, peripheral vascular disease

## Abstract

There are various physical signs that can be used as a reliable tool to diagnose the subclinical stages of atherosclerosis, including corneal arcus, xanthelasma, and diagonal earlobe crease (DELC) or “Frank's sign”. Bilateral diagonal earlobe crease has been positively correlated with coronary artery disease (CAD) and peripheral vascular disease (PVD). The presence of DELC has been identified as an independent variable for CAD. The exact etiology is unknown and the association between the presence of DELC and coronary artery disease (CAD) still remains controversial. We report a case of a patient with bilateral DELC who was found to have remarkable non-occlusive CAD on diagnostic coronary angiography.

## Introduction

Frank's sign is a diagonal earlobe crease (DELC) which is a wrinkle that extends 45° backward from the tragus to the auricle; it is hypothesized to be a predictor of atherosclerotic disease. This sign has been positively correlated with coronary artery disease (CAD) and peripheral vascular disease (PVD) [1-3] and was classified as an independent variable for CAD [4-5]. This correlation was first described in 1973 by an American physician, Sanders T. Frank [6]. The etiology is unclear; some studies suggested a parallel process of age-related and microvascular disease associated weakening of elastic fibers of earlobes and in coronary arteries [7].

## Case presentation

**Figure 1:**
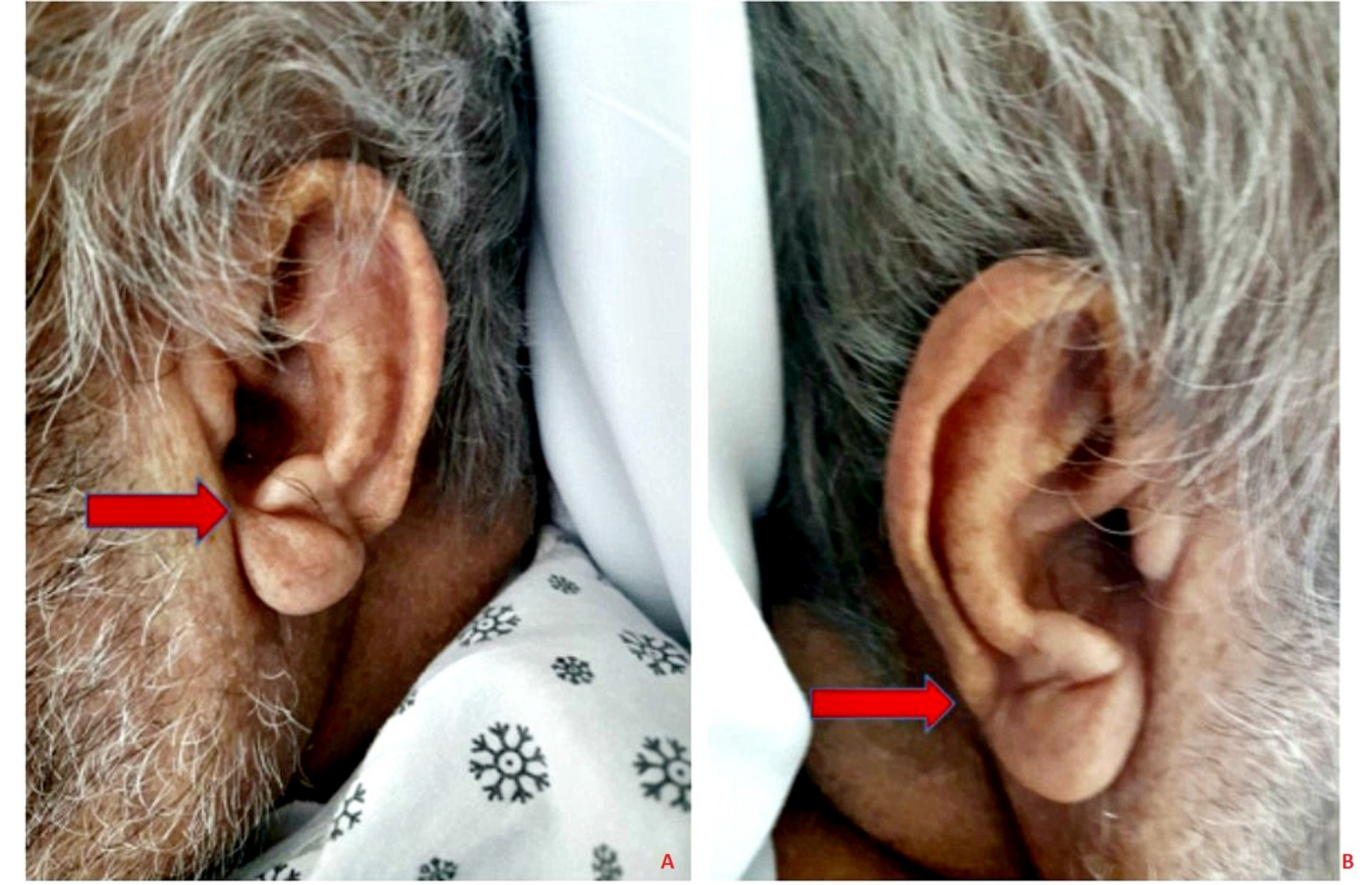
Bilateral diagonal earlobe creases as indicated by arrows

**Figure 2:**
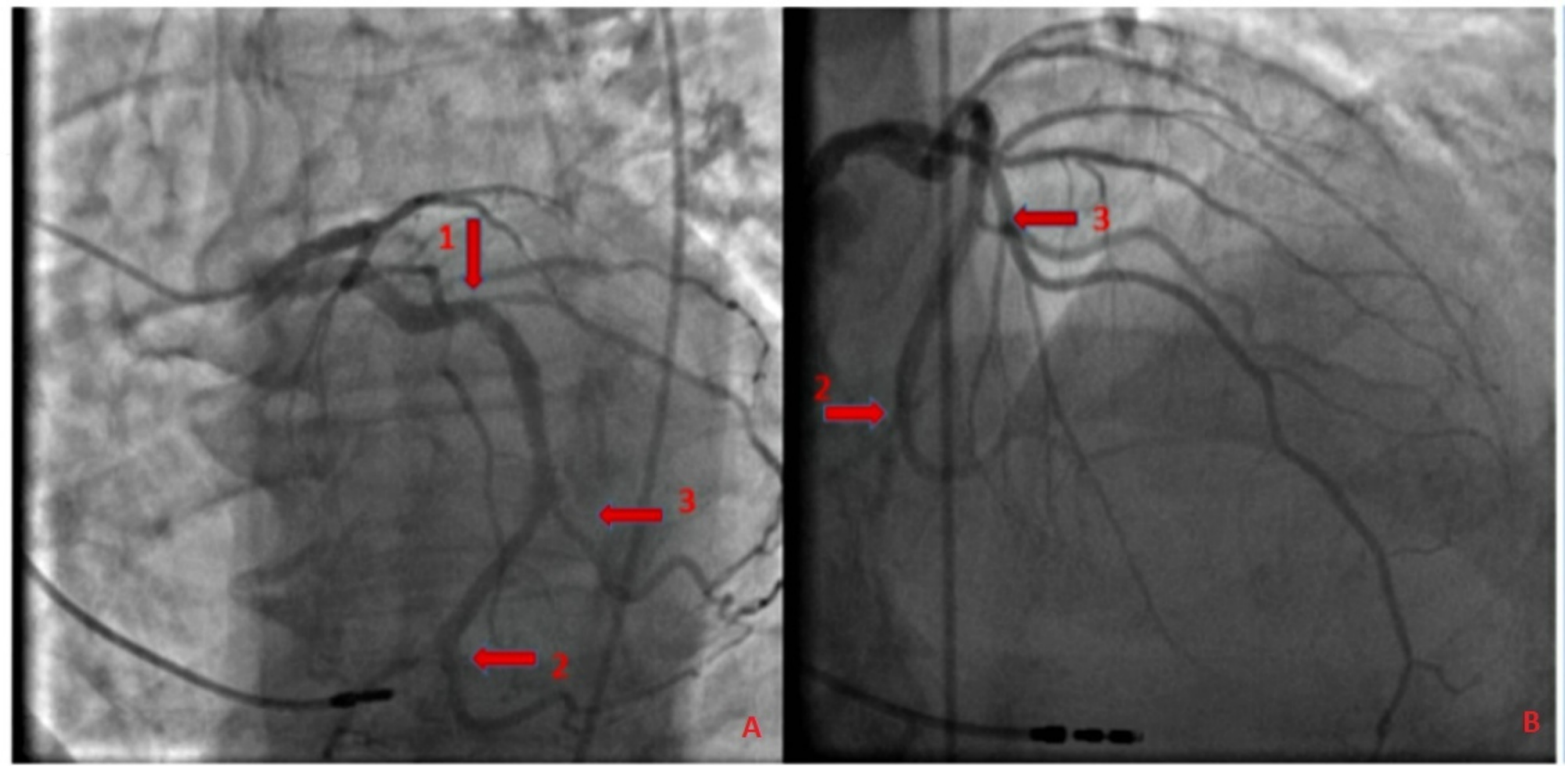
Coronary angiogram Panels A and B: Coronary angiogram showing disease of the circumflex artery - 30% lesion in the proximal segment (arrow 1), a 50% lesion in the distal segment (arrow 2), and a 50% lesion in the proximal segment of the obtuse marginal artery (arrow 3).

An 83-year-old male ex-smoker presented with progressive worsening of shortness of breath and dry cough for three days. His past medical history was significant for sick sinus syndrome, status-post pacemaker placement in 2014, heart failure with a reduced ejection fraction (HFrEF), hypertension, diabetes mellitus with dermopathy, rectal cancer status-post colostomy, chronic obstructive pulmonary disease (COPD), benign prostatic hypertrophy, and gout. He reported associated rhinorrhea and malaise and had been using his albuterol inhaler at home with no relief. He denied any associated chest pain, palpitations, pre-syncope, or syncope. Physical examination was remarkable for bilateral DELC, more prominent in the left ear (Figure 1). Vital signs were stable. An electrocardiogram (EKG) showed an atrial paced rhythm at 89 beats per minute. A chest x-ray revealed clear lung fields with minimal atelectasis. Labs were pertinent for elevated troponin and D-dimer; troponin peaked at 0.58. He was started on a heparin drip. He underwent computed tomography angiogram (CTA) of the chest which ruled out a pulmonary embolus. A transthoracic echocardiogram revealed an ejection fraction of 35% - ­40%, Grade 1 left ventricular diastolic dysfunction, and abnormal septal motion consistent with a conduction abnormality. He subsequently underwent a coronary angiogram which revealed disease of the circumflex artery with a 30% lesion in the proximal segment, a 50% lesion in the distal segment, and a 50% lesion in the proximal segment of the obtuse marginal artery focal lesion) (Figure 2). In view of the presence of non-obstructive CAD, he was started on appropriate medical therapy and discharged with a cardiology follow-up for medical optimization.

## Discussion

Diagonal earlobe crease (DELC), the wrinkle at 45° between the auricle and the tragus, also known as Frank’s sign, was first reported to be associated with CAD by Sanders T. Frank in 1973 when he noted it in 20 patients with angina [6]. From the time of its first description, studies have reported an association of DELC with the prevalence, extent, as well as the severity of CAD, and the risk of adverse cardiac events independent of commonly known risk factors, such as hypertension, diabetes, hyperlipidemia, and smoking [3-5], with bilateral complete DELC being the most severe.

Among these studies was the Copenhagen City Heart Study, a 35-year-long prospective study that reported earlobe crease, xanthelasma, and frontotemporal/crown top baldness for being independently associated with ischemic heart disease and myocardial infarction after multifactorial adjustment for well-known cardiovascular risk factors [8]. A study reported by Pasternac and Sami was conducted to evaluate the value of DELC in predicting the presence of coronary artery disease by examining the earlobes of 340 patients who underwent coronary arteriography for variable cardiac complaints. They reported that in 75.6% of those patients who had coronary artery disease, the sensitivity of the sign was 59.5%, the specificity 81.9%, and the positive predictive value was 91.1% [9]. The etiology of this association is unclear; some studies suggested a parallel process of age-related and microvascular disease associated weakening of elastic fibers of earlobes and in coronary arteries. 

## Conclusions

Our patient demonstrated bilateral earlobe creases in the setting of remarkable non-occlusive CAD. Recognition of this easily detectable sign by clinicians may facilitate prompt evaluation and early diagnoses of coronary atherosclerotic disease, especially in the presence of other concurrent risk factors, and help patients adapt a healthier lifestyle to help prevent the onset and progression of coronary disease.
